# Special Issue “Advances in Antiviral Agents Against SARS-CoV-2 and Its Variants” 2nd Edition

**DOI:** 10.3390/v18010059

**Published:** 2025-12-30

**Authors:** Francesca Esposito

**Affiliations:** Department of Life and Environmental Sciences, University of Cagliari, Cittadella Universitaria SS554, 09042 Monserrato, Italy; francescaesposito@unica.it

Since the emergence of severe acute respiratory syndrome coronavirus 2 (SARS-CoV-2), extensive efforts have been undertaken to identify effective therapeutic strategies to prevent and treat coronavirus disease 2019 (COVID-19). While vaccines and a limited number of direct-acting antivirals have substantially reduced severe disease and mortality, the continuous emergence of viral variants, disparities in global access to therapies, and the burden of post-acute sequelae of COVID-19 underscore the need for improved and broadly applicable antiviral strategies. This Special Issue of *Viruses* brings together original research articles and a comprehensive review that critically examine drug repurposing, host-directed therapies, combination treatments, preclinical infection models, and alternative antiviral approaches, providing an integrated perspective on their translational potential and limitations.

A central contribution to this Special Issue is the study by Hurwitz et al. [1], which addresses the fundamental reasons why most repurposed drugs have failed to demonstrate clinical efficacy against COVID-19. By systematically evaluating the antiviral activity (EC_50_) and cytotoxicity (CC_50_) of 49 candidate compounds and integrating these data with reported unbound peak plasma concentrations, the authors demonstrate that many drugs progressed to clinical trials without achieving sufficient exposure relative to antiviral potency. Importantly, compounds showing clinical benefit exhibited unbound Cmax/EC_50_ ratios ≥ 6.8 in relevant human respiratory cell models. This work highlights the critical importance of pharmacokinetic–pharmacodynamic relationships and appropriate cellular systems, illustrating how reliance on non-representative in vitro models has contributed to numerous unsuccessful clinical trials.

In contrast, other contributions demonstrate that repurposing can still represent a viable strategy when supported by strong mechanistic rationale and rigorous experimental validation. Thümmler et al. [2] report that the antidepressants fluoxetine and sertraline, functional inhibitors of acid sphingomyelinase, potently inhibit replication of multiple SARS-CoV-2 variants, including D614G, Alpha, Delta, and Omicron sub-lineages BA.1 and BA.5, in vitro. The broad antiviral activity observed across genetically diverse variants suggests that host-directed therapies may offer increased resilience to viral evolution and supports further investigation of these compounds in well-designed clinical studies.

The importance of relevant in vivo models is emphasized by studies employing the Syrian hamster model of SARS-CoV-2 infection. Generalova et al. [3] demonstrate that treatment with the polysaccharide-based compound Immeran significantly suppresses viral replication in the lungs and stimulates the production of virus-neutralizing antibodies, indicating both antiviral and immunomodulatory properties. Complementarily, Abdelnabi et al. [4] compare the infectivity and pathogenicity of recent Omicron sub-variants, showing that EG.5.1 and BA.2.86 exhibit attenuated lung replication and reduced pathology compared with BA.5. These findings provide important context for the evaluation of updated vaccines and therapeutic strategies targeting emerging variants.

Combination antiviral therapy is explored by Fiaschi et al. [5], who assess the in vitro activity of licensed direct-acting antivirals and monoclonal antibodies against ancestral and divergent SARS-CoV-2 variants. Although most combinations resulted in additive effects, concentration-dependent cooperative interactions were identified, particularly among antiviral–antiviral combinations. These data provide a rational basis for further investigation of combination regimens in preclinical models prior to clinical translation.

Finally, the comprehensive review by Al-Jamal et al. [6] expands the therapeutic landscape by critically evaluating the potential role of medicinal plants and plant-derived bioactive compounds in COVID-19 treatment. By summarizing available evidence on the antiviral and immunomodulatory activities of selected plant species, this review highlights both the promise and the substantial challenges associated with translating traditional herbal remedies into evidence-based antiviral therapies.

Collectively, the contributions to this Special Issue provide a balanced and critical overview of current and emerging approaches to SARS-CoV-2 treatment. They emphasize that successful antiviral development requires rigorous pharmacological evaluation, appropriate experimental and animal models, and careful consideration of translational relevance. Beyond COVID-19, the insights presented here offer valuable lessons for improving preparedness and therapeutic responses to future emerging viral infections.
